# Sporadic incidence of Fascioliasis detected during Hepatobiliary procedures: A study of 18 patients from Sulaimaniyah governorate

**DOI:** 10.1186/1756-0500-5-691

**Published:** 2012-12-21

**Authors:** Tahir Abdullah Hussein Hawramy, Kamal Ahmed Saeed, Seerwan Hama Sharif Qaradaghy, Taha Ahmed Karboli, Beston Faiek Nore, Noora Hisham Abood Bayati

**Affiliations:** 1 Sulaimani Teaching Hospital, Dept. of General Surgery, Sulaimaniyah, Kurdistan Region, IRAQ; 2 Dept. of General Surgery, School of Medicine, Faculty of Medical Sciences, University of Sulaimani, Sulaimaniyah, Kurdistan Region, IRAQ; 3 Kurdistan Center of Gastroenterology and Hepatology (KCGH), Sulaimaniyah, Kurdistan Region, IRAQ; 4 Department of Internal Medicine, School of Medicine, Faculty of Medical Sciences, University of Sulaimani, Sulaimaniyah, Kurdistan Region, IRAQ; 5 Dept. of Biochemistry, School of Medicine, Faculty of Medical Sciences, University of Sulaimani, Sulaimaniyah, Kurdistan Region, IRAQ

**Keywords:** Fasciola hepatica, *Fasciola gigantic*a, Zoonotic disease, Hepatobiliary surgery, Flukes, Stone analysis, ERCP

## Abstract

**Background:**

Fascioliasis is an often-neglected zoonotic disease and currently is an emerging infection in Iraq. Fascioliasis has two distinct phases, an acute phase, exhibiting the hepatic migratory stage of the fluke’s life cycle, and a chronic biliary phase manifested with the presence of the parasite in the bile ducts through hepatic tissue. The incidence of Fascioliasis in Sulaimaniyah governorate was unexpected observation. We believe that shedding light on this disease in our locality will increase our physician awareness and experience in early detection, treatment in order to avoid unnecessary surgeries.

**Findings:**

We retrospectively evaluated this disease in terms of the demographic features, clinical presentations, and managements by reviewing the medical records of 18 patients, who were admitted to the Sulaimani Teaching Hospital and Kurdistan Centre for Gastroenterology and Hepatology. Patients were complained from hepatobiliary and/or upper gastrointestinal symptoms and diagnosed accidentally with Fascioliasis during hepatobiliary surgeries and ERCP by direct visualization of the flukes and stone analysis. Elevated liver enzymes, white blood cells count and eosinophilia were notable laboratory indices. The dilated CBD, gallstones, liver cysts and abscess were found common in radiological images. Fascioliasis diagnosed during conventional surgical CBD exploration and choledochodoudenostomy, open cholecystectomy, surgical drainage of liver abscess, ERCP and during gallstone analysis.

**Conclusion:**

Fascioliasis is indeed an emerging disease in our locality, but it is often underestimated and ignored. We recommend the differential diagnosis of patients suffering from Rt. Hypochondrial pain, fever and eosinophilia. The watercress ingestion was a common factor in patient’s history.

## Findings

### Background

Human Fascioliasis is a zoonotic disease caused by the trematodes of genus *Fasciola,* most commonly in two species *Fasciola hepatica and Fasciola gigantica*[1]. World health organization now recognizes human Fascioliasis as a significant public health problem and *a neglected tropical disease* with a great impact on human development [2]. The life cycle of this parasite starts when eggs in mammalian stool are deposited in tepid water (22-26°C), miracidia appear, develop, and hatch in within two weeks. These miracidia invade many species of freshwater snails, in which they further develop to sporocyst and redia for 4-7weeks. They leave as free-swimming single tailed cercaria that subsequently attach to watercress, water lettuce, mint, parsley, or khat [3,4]. They encyst within few hours and wait to be eaten by the definite host, humans [2]. The metacercariae exist in the small intestine is releasing the young parasites, which then rapidly penetrate the intestinal wall and enter the peritoneal cavity. The immature flukes penetrate the capsule of Glisson after 48h and enter the liver then migrate throughout the hepatic parenchyma till they reach the biliary system where they become adults within 3 to 4 months from the initial infection and lay eggs [2].

The clinical presentation of Fascioliasis depends on two different phases of the infection. The acute or hepatic phase occurs when the worm migrates through the liver parenchyma and can last for up to 3 months after ingestion of metacercariae. Eosinophilia, hepatomegaly, ascites, subscapular hemorrhage, hepatic necrosis, hepatic abscesses and pulmonary infiltration have also been described in this phase [5]. The second phase is the chronic or biliary phase, which begins when the adult flukes enter the biliary tree, where they can remain asymptomatic for many years. They occasionally cause inflammation, epithelial hyperplasia and fibrosis, which can lead to biliary obstruction, cholangitis, pancreatitis [6] and hemophilia [5]. Liver masses or abscess and anemia may be present [7].

To diagnose human Fascioliasis several techniques are available; direct parasitological examination for fecal sample and biliary aspirate for the presence of eggs or rarely the parasite. Intradermal test and stool antigen detection tests are also widely used; they provide the advantage of being applicable during all stages of disease and are useful to monitor post-treatment evolution . Radiological techniques also provide diagnostic tools like ultrasonography, CT scan, MRI and MRCP. Endoscopic retrograde cholangiopancreatogram (ERCP) is increasingly used to diagnose and treat human Fascioliasis particularly in the biliary stage [5]. *Fasciola hepatica* infestation had been diagnosed accidentally during hepatobiliary surgeries [8] and ERCP [5,9-11]. Here in this study we are presenting a similar situation. Triclabendazole is the drug of choice for the treatment of both the acute and chronic phases. The recommended dose is 10mg/ kg as single dose taken with fatty meal for better absorption. The dose can be repeated 12hr or 24hr apart for severe cases, while Albendazole and Prazyquentil have been used with variable degrees of success [2].

In Iraq, the first case of Fascioliasis was reported in 1964. It was an ectopic human Fascioliasis in the eye by an immature *Fasciola gigantica* worm [12]. In 2004, we reported the first case of biliary Fascioliasis, which was diagnosed accidentally during CBD exploration [13]. Ezzat et al. described for the first time a successful endoscopic ERCP management [14]. In 2010, we reported a case of an adult *Fasciola hepatica* worm found inside the gallbladder during a conventional Cholecystectomy [15]. In this report, we shade light on this neglected disease hoping to induce our clinician and global awareness. We give recommendation to the clinicians in our community to introduce precise serology tests to solve many of the misdiagnoses problems and so avoiding unnecessary surgeries.

### Patients and method

A retrospective study was conducted on 18 patients who were admitted to the Sulaimani Teaching Hospital and Kurdistan Centre for Gastroenterology and Hepatology from 1997 to march 2012. To conduct this study, ethical permission was approved from Ethics Committee at School of Medicine, Faculty of Medical Sciences, University of Sulaimani). Patients were complaining from hepatobiliary and/or upper gastrointestinal symptoms and later diagnosed accidentally with Fascioliasis during the course of their management. The data collected from the medical records of these 18 patients; covering history, physical examinations and laboratory investigations for hemoglobin level, alkaline phosphatase, GOT, GPT, total serum bilirubin, and white blood cells count, but in some cases eosinophil count and general stool examination were performed. For all cases informed consents were obtained. Abdominal imaging was taken randomly for some patients, either with ultra sonogram or endoscopic ultra sonogram (EUS) or computerized tomography (CT), or magnetic resonant cholengiopancreatogram (MRCP). The definite diagnosis of Fascioliasis was made by direct visualization of the adult worms during conventional hepatobiliary surgeries and during ERCP and confirmed by parasitological examination. Gall stone analysis showed calcified worm in 2 cases.

## Results

Our patients were 15 female and 3 males (female: male ratio is 5:1). They were aged from 25 years to 82 years with a mean of 42.5 years. Only six patients (33.3%) were urban inhabitant, while 12 patients (66.6%) were from rural areas of Sulaimaniyah governorate. Fourteen patients (77.7%) had a history of raw watercress ingestion.

Right upper abdominal pain was the main cause for admissions in 16 patients (88.8%) and it was associated with tenderness in 66.6 % of the patients. Malaise, fever, rigor, dyspepsia, jaundice, nausea and vomiting and anemia were recorded (Table 1). Laboratory data analysis showed that 72.2% of the patients (n=13) had elevated liver enzymes (e.g. alkaline phosphatase, GPT, GOT). Hyperbilirubinemia was found in 44.4% (n=8) of the registered cases. Elevated white blood cells count was again observed in 55.5% (n=10) of the patients, while eosinophilia detected in 100% of the eight patients, who had this test. Stool examination for ova detection was done in 4 of the cases postoperatively, but only one case found positive. With imaging, the most common radiological abnormality was dilated CBD observed in 16 patients (88.8%). We also recorded CBD stone, gallstones, hepatomegaly, cystic lesions and liver abscess (Table 2). The most common provisional diagnoses were cholangitis, cholecystitis, biliary colic, liver abscess and post cholecystectomy jaundice (Table 3). Finally, the clinical data and symptom durations gave indications that 3 patients were in the acute stage of the infestation (16.6%), while 15 patients were in the chronic stage (83.3%) (Table 3).

**Table 1:** Frequency of presenting symptoms

**Presenting symptoms**	**No. of patients**	**Percentage (%)**
RUQ pain	16	88.8%
RUQ tenderness	12	66.6%
Malaise	11	61.1%
Fever and rigor	10	55.5%
dyspepsia	9	50%
jaundice	5	27.7%
Nausea and vomiting	6	33.3%
pruritis	3	16.6%
Epigastric pain	2	11.1%
anemia	2 (Hg less than 9g/dl)	11.1%

**Table 2:** Frequency of imaging study abnormalities

**Imaging abnormality**	**No. of patients**	**Percentage (%)**
Dilated CBD	16	88.8%
CBD stone or shadow	9	50%
Hepatomegaly	6	33.3%
Liver abscess	3	16.6%
Gall stone	2	11.1%
Hepatic cystic lesions	1	5.5%

**Table 3:** Frequency of the presenting disease

**Provisional diagnosis**	**No. of patients**	**Percentage (%)**
Cholangitis	7^*^	38.8%
Calculous cholecystitis	3	16.6%
Biliary colic	4	22.2%
Liver abscess	3	16.6%
Postcholecystectomy jaundice	1	5.5%

*post cholecystectomy n=3.

Since *Fasciola hepatica* was not known until recent years in our country, therefore the diagnoses were done incidentally by direct visualization of the adult worms during conventional hepatobiliary surgeries (8 cases), or during ERCP (10 cases) and/or stone analysis (2 cases) which showed calcified parasite upon parasitological examination (Table 4). Five of ERCP cases were post cholecystectomy and one case associated with Ultrasound guided percutaneous drainage of liver abscess. The treatment modalities were recorded and analyzed. Eight patients (44.4%) received Albendazole 400 mg t.d.s. for 1 week postoperatively. Only 3 cases (16.6%) received single dose of Triclabendazole 10 mg/kg and seven patients (38.8%) didn’t receive any anthelminthic drugs postoperatively.

**Table 4:** Method of intervention

**Provisional diagnosis**	**No. of patients**	**Percentage (%)**
Cholecystectomy + CBD exploration + Choledochodoudenostomy	4	22.2%
* Cholecystectomy	*2	11.1%
Cholecystectomy + Drainage of liver abscess or cyst	2	11.1%
* ERCP	*10	55.5%

* One cases of stone analysis.

## Discussion

Although Fascioliasis is endemic in our neighboring countries (Turkey and Iran), but it seems to be an emerging health problem in our locality and also in whole Iraq. We retrospectively reviewed and analyzed all the cases that had been diagnosed with Fascioliasis in our hospital during the past 15 years. Abdominal pain was the most common presentation and the predominant cause of admission, mostly at Rt. Hypochondrial region in 16 cases (88.8%) and association with tenderness in 12 cases (66.6%). Malaise was recorded in (61.1%) of patients. More than half of our patients (55.5%, n=10) described attacks of fever and rigor, which may be either due to hepatic abscess formation during the 1^st^ hepatic phase of the disease or due to cholangitis at the 2^nd^ ; biliary phase. Dyspepsia, which usually present during the acute phase was present in 50 %. Six patients (33.3%) got nausea and infrequent vomiting. Long-standing anemia (Hg level is less than 9g/dl) was recorded in 2 cases.

The laboratory diagnosis was requested to evaluate the cases with hepatobiliary problems, although Fascioliasis was not the suspected cause at the time of admission. Almost three quarters of our patients had elevated serum Alkaline phosphatase (72.2%, n=13) and similar findings were reported [16]. It is known that elevated liver enzymes especially alkaline phosphatase is one sign of liver invasion by the parasite [2].

In our study leukocytosis was detected in 10 patients, but only 5 patients had differential WBC, which showed eosinophilia in all of them. This observation should alert our physicians to ask for differential WBC counts rather than the total count, which may highlight a parasitic infestation in the differential diagnosis of hepatobiliary problems. Stool examination for egg detection was done for 4 patients; only 1 patient had positive results. The presence of the *Fasciola* eggs in the stool early in the course of the disease is one useful way to confirm the diagnosis, but repeated stool collections are recommended [17].

In Cosme et al. study [16], egg detected in feces was only found positive in 6 cases out of 37, but in Sierra et al. study [18], 221 patients were found positive for eggs out of 278 patients. On the other hand, duodenal sondage (fluid aspirate) was performed in 90 patients, yielding egg detection in 67. Interestingly, nine of them appeared negative in a stool examination, while positive in duodenal sondage [19]. This reveals that stool examination as non-reliable test.

Imaging studies of the hepatobiliary system with ultrasonography and CT scan can sometimes be useful in establishing a diagnosis with variable sensitivity for detecting hepatic abnormalities, like parenchymal heterogeneity and/or multiple small cystic hepatic lesions or tortuous structures. In chronic phase can show motile flukes inside the gallbladder. Ultrasound guided gallbladder aspiration is a new diagnostic method for biliary Fascioliasis and it is safe, less invasive, and also can show the presence of eggs in the bile [2,10].

In our study, abdominal ultrasonography was the imaging modality most commonly used for all of the 18 patients and dilated CBD was detected in 16 of them (88.8%). However, 9 cases were in association with stone or a shadow inside the CBD. This observation was also present in Cosme et al. [16] patients indicating chronicity of the infestation. Gallstones, Liver abscess and liver cysts were also detected in our patients, who were also recorded in other reports [2,9,16,18].

In many human case reports, serology was the sole diagnostic method [20], but unfortunately, none of our cases was sent for serology testing for Fascioliasis because it was not available in our province at time of cases presentations. Other used techniques that aid the diagnosis are, (1) liver scan using ^99m^Tc uptake, which may show irregular radio colloid and/or lacunar areas. (2) Laparoscopy allows viewing features of diseased liver with tunnel like lesions on its surface and especially yellow-white nodules with a halo hyper-vascularized of different sizes and shapes. (3) Other injuries to the Glisson capsule, peritoneum and liver biopsy can be taken for diagnosis at the same time [16]. Histological features of Fascioliasis demonstrate granulomas, which should be differentiated from other well-recognized diseases including tuberculosis, sarcoidosis, toxoplasmosis, and non-Hodgkin’s lymphoma. The most typical histological finding is central necrosis, cellular debris, and Charcot-Leyden crystals encompassed by eosinophil and inflammatory infiltrate.

In 4 patients, CBD exploration was done for prominent dilatation associated with jaundice. Live *Fasciola* worms were found to cause this dilatation and obstructing the bile flow. Removal of the flukes performed followed by Choledochoduodenostomy and Cholecystectomy.

One case presented with chronic calculus cholecystitis symptoms and ultrasound showed a single gallstone and normal biliary tree. For this patient, open Cholecystectomy was done and the stone was sent for analysis. Interestingly a calcified *Fasciola hepatica* worm was forming the nidus for the gallstone. Indeed, gallstone disease has recently proved to be one of the effects of advanced chronic Fascioliasis, since *Fasciola hepatica* is able to survive up to 9–13.5 years within a human host [18]. Two other cases were diagnosed with Liver abscesses. Eventually, they underwent abdominal exploration for drainage and live worms were found in the abscesses cavities. This indicates that these two patients were in the acute stage of the disease.

**Figure 1:**
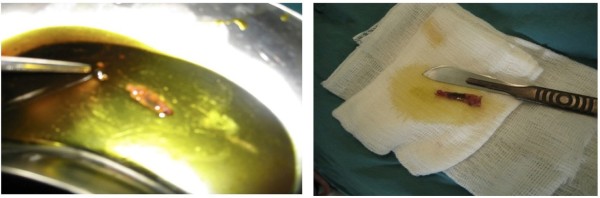
A case of conventional cholecystectomy (A) Fasciola worm swims in the bile in vitro (B) Fasciola worm in vitro.

In another case, a patient was admitted for recent features of calculus cholecystitis. Hence open cholecystectomy was done and upon opening the gallbladder in vitro a live motile *Fasciola* was found swimming inside the gallbladder (Figure 1 ). The patient Rt. hepatic lobe also had 2 small cystic lesions that were aspirated and sent for parasitological study. The aspirate was clear and negative for the eggs.

Facing Fascioliasis during surgery or ERCP is a recognized issue worldwide, but it is obscure in our country. It is worthwhile to mention that cases admitted before 2006 were found positive only during conventional hepatobiliary surgery. Since ERCP was introduced to our hospital on 2006, cases afterword was discovered endoscpoically.

**Figure 2:**
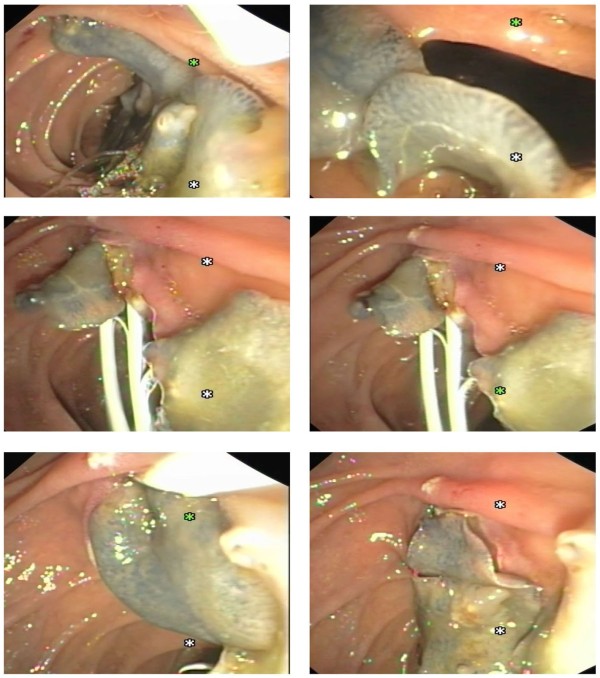
In another patient, 14 live Fasciola hepatica worms escaped from C.B.D. during endoscopic sphincterotomy / ERCP.

When ERCP became available in our province, the first case of biliary tree Fascioliasis was diagnosed and treated*.* The patient presented 6 months post laparoscopic cholecystectomy with obstructive jaundice. The ERCP with sphinecterotomy managed to retrieve a live fluke from the CBD (Figure 2). Notably, another 4 cases of post cholecystectomy obstructive jaundice were diagnosed within 6–9 months postoperatively and managed similarly. Another case of obstructive jaundice, Rt. hypochondria pain and pruritus (not cholecystectomis) was diagnosed to have biliary Fascioliasis and managed by ERCP. Indeed intraoperative cholangiography would have been helpful in diagnosing CBD infestation with the flukes. On the other hand, these cases could have been in the acute stage, when they underwent the cholecystectomy and the flukes invaded the biliary tree within the 6-9months postoperatively.

A later patient presented with liver abscess, confirmed by CT scan and managed by ultrasound guided percutaneous drainage (using pigtail catheter) with daily aspiration and irrigation of the cavity. It was completely empty of pus in conjugation with ERCP with sphincterotomy and removal of a live *Fasciola hepatica* from the CBD. Then another case managed by ERCP, was presented 17 years post open cholecystectomy with obstructive jaundice. Ultrasound showed a large CBD stone. Utilizing endoscopic mechanical lithotripsy, stone fragments were collected and sent for analysis. Surprisingly a calcified *Fasciola hepatica* worm was found with the fragments. Similar situation was reported, Obstructive jaundice due to biliary *Fasciola hepatica* infestation observed 11 years post-open cholecystectomy and CBD exploration [21]. Another interesting case of biliary fascioliasis was diagnosed by retrieving 14 live adult worms from the CBD (video) the last two cases presented with anemia and abdominal pain and ERCP retrieved *Fasciola* flukes from CBD. Indeed anemia is recorded in other studies as associated symptom and sign [22].

Fascioliasis could be completely cured using Triclabendazole as first choice anthelminthic. Triclabendazole is a Benzimidazole compound used routinely in veterinary practice since 1983 for treatment of Fascioliasis, but it was not used in humans until the 1989, when the Iranian authorities approved the use of the veterinary formulation to treat human epidemic of Fascioliasis near the Caspian Sea. The recommended dose is 10-mg/kg body weights as single dose administered with food containing fat to increase the absorption. A second dose can be given 12 hrs. intervals for severe cases [2]. Only three of our cases received a single dose of triclabendazole, obtained from outside Iraq. The other eight patients received Albendazole 400 mg t.d.s. for 7 days postoperatively. The remaining patients denied any drug administration. Still, we are facing difficulty in obtaining Triclabendazole and the same situation is true for Cosme et al. [16] and Sierra et al. [18]. For decades; Emetine and dehydroemetine were used successfully to treat Fascioliasis with a cure rate reaching 90%, but it has toxic side effect on heart, liver and digestive tract.

## Conclusion

We are documenting the presence of Fascioliasis in Sulaimaniyah province in Kurdistan Region - Iraq. Fascioliasis is an emerging disease in our locality, but it is underestimated and ignored. It should be considered strongly in cases of hepatic abscesses and obstructive jaundice and in the differential diagnosis of Rt. Hypochondrial pain, fever and eosinophilia. Clinicians should request for differential WBC count to identify eosinophilia, as 100% of infected individuals suffer from eosinophilia. We should introduce precise serology tests that solve many of the misdiagnoses problems avoiding unnecessary surgeries as drug treatments completely cure the disease. Public awareness about this zoonotic disease and its effect on human and livestock is of high importance, especially the role of aquatic plants in transmitting Fascioliasis.

### Consent

Patients and patient families were informed and a written consent was obtained. A copy of these consent documents is available for review by the Editor-in-Chief of this journal.

## Abbreviations

ERCP: Endoscopic retrograde cholangiopancreatography; CBD: Common Bile Duct; WHO: World health organization; CT: Computed tomography; EUS: Endoscopic ultrasound; MRI: Magnetic Resonance Imaging; MRCP: Magnetic resonance cholangiopancreatogram; GOT: Glutamic-oxaloacetic transaminase; GPT: Glutamic-pyruvic transaminase.

## Competing interests

The authors declare no competing financial interests.

## Authors’ contributions

TAHH designed the project and coordinated the overall project. TAHH performed the conventional hepatobiliary surgeries, evaluated, and followed up them. NHAB followed up the patients, gathered data, analyzed and wrote the manuscript. TAK and HA performed the ERCP, EUS and evaluated the endoscopic cases. KAS and SHSQ supervised NHAB to work with this project. BFN contributed in writing, editing and restructuring the style of the manuscript, including references and also presenting most the scientific formulations. All authors read and approved the manuscript.
